# The impact of dysfunctional tear films and optical aberrations on chronic migraine

**DOI:** 10.1186/s40662-017-0070-1

**Published:** 2017-02-21

**Authors:** Rohit Shetty, Kalyani Deshpande, Chaitra Jayadev, Kareeshma Wadia, Pooja Mehta, Rushad Shroff, Harsha L. Rao

**Affiliations:** Department of Ophthalmology, Narayana Nethralaya Eye Institute, 121/C, Chord Road, 1 “R” Block, Rajajinagar, Bangalore, 560010 Karnataka India

**Keywords:** Migraine, Dry eye, Aberrations

## Abstract

**Background:**

Migraine is a multifactorial disorder with complex neuronal and vascular mechanisms that encompasses a wide clinical spectrum of symptoms, including ocular manifestations. Dry eye disease and dysfunction of ocular somatosensory pathways have been implicated in the pathogenesis. The current study investigates the association between a dysfunctional tear film and ocular aberrations with migraine.

**Methods:**

Sixty eyes of 30 patients with migraine and 60 eyes of 30 controls were studied. Dry eye evaluation included Schirmer’s test, tear film break-up time, corneal esthesiometry and lipid layer analysis using Lipiview® interferometer. Wavefront aberrations were measured using Optical Path Difference before performing the dry eye evaluation. The intraocular light scatter was quantified using the objective scatter index (OSI) of the optical quality analysis system. Measured parameters were compared between the migraine and the control group using independent sample *t*-test. Statistical analysis was performed using commercial software. A *p* value of ≤ 0.05 was considered statistically significant.

**Results:**

There were 19 females and 11 males in each group. Statistically significant difference was found between the two groups with respect to total aberrations (*p* = 0.049), higher order aberrations (*p* = 0.009), coma (*p* = 0.03), spherical aberrations (*p* = 0.018), Lipiview interferometric coloric units (*p* < 0.001) and OSI (*p* < 0.001). Trefoil (*p* = 0.26) and TBUT (*p* = 0.398) were not significantly different between both groups.

**Conclusions:**

Ocular aberrations are higher in patients with migraine as compared with controls. Tear film abnormalities add to the aberrations in otherwise asymptomatic patients and may also be associated with migraineous attacks. Treating the ocular surface to obtain a healthy tear film might introduce a potential modifiable factor in the prevention of migraneous attacks.

## Background

The relationship between the eye and migraine is an interesting one. Ocular effects of migraine involve the eyelids, pupil, optical nerve, retina, extraocular muscles and migraneous ocular pain [1]. The most common migraine aura is visual in nature [2]. Undiagnosed migraineurs may first present for an eye examination looking for answers to their ocular symptoms [3], including photophobia or intolerance to light, which are also seen in other ocular diseases [4–6].

The tear film is the first and most important refractive surface of the eye [7]. An unhealthy ocular surface can lead to increased tear film break-up and hence result in image degradation secondary to increased wavefront aberrations [8]. Imperfections in the optical system that occur due to irregularities of the optical surfaces and differences in the refractive indices are called aberrations. Changes in the visual environment that lead to evaporative dry eye and alterations in the ocular surface may influence aberrations. Dry eye disease is also noted to be more frequent in migraine patients with a greater presence of aura, longer duration of attacks and longer disease durations [9]. The trigeminal nerve is the common afferent nerve for the migraine pathway as well as for dry eye associated ocular hyperalgesia [10, 11]. It is therefore interesting to explore the role of eye disease in aggravating the migraine symptoms. The current study looks at the correlation between migraine and a dysfunctional tear film as well as ocular aberrations in migraine patients as well as controls.

Chronic migraine is characterized by headaches occurring for 15 or more days in a month [12]. With a prevalence of 0.5-2%, the conversion to chronic migraine is approximately 2.5% every year [12, 13]. Several factors may be responsible for this negative evolution in the course of migraine and hence it is worthwhile to identify preventable or reversible factors. Hence, the current study investigates the role of a dysfunctional tear film and ocular aberrations in migraine by comparing ocular aberrations, ocular scatter index (OSI) and the tear film break-up time (TBUT) between controls and those with migraine.

## Methods

This non-interventional, observational cross sectional study was approved by the hospital’s institutional review board and was in agreement with the tenets of the Declaration of Helsinki. All patients after having been given a full explanation of the procedure provided a written informed consent. Subjects aged 18–35 years, spherical equivalent (SE) within ± 0.50 D without systemic diseases associated with dry eye (e.g., rheumatoid arthritis), any pre-existing ocular disorder or history of ocular surgery served as controls. Patients with a confirmed diagnosis of chronic migraine with aura according to international classification of headache disorders were included in the study after neurological consult.

After a thorough clinical history to rule out ocular and systemic co-morbidities, visual acuity assessment, refraction, detailed slit-lamp examination and fundus evaluation were performed. Subjects were tested between 10:00 AM and 1:00 PM in a dimly lit consulting room under ambient conditions of temperature and humidity. Schirmer’s test without anesthetic (Schirmer’s I) was performed using sterile Schirmer’s strips - Whatman filter paper measuring 5 × 35-mm^2^ (Contacare Opthalmics and Diagnostics, India) to rule out aqueous deficiency dry eye disease. A hanging drop of 1% fluorescein stain (Contacare Opthalmics and Diagnostics, India) was instilled in the conjunctival cul-de-sac to note the TBUT with corneal and conjunctival epithelial staining if any. Corneal sensations were assessed in all patients with the Cochet-Bonnet esthesiometer (Luneau ophthalmologia, Chartes, France). The lipid layer of the tear film was objectively measured and quantified in interferometric coloric units (ICU) using the Lipiview® interferometer (TearScience, Morrisville, N.C). Wavefront aberrations in root mean square (RMS) were measured using the mesopic wavefront by the Optical Path Difference (OPDIII, Nidek, Japan) before performing the dry eye evaluation mentioned above. The intraocular light scatter was quantified using the OSI of the optical quality analysis system (OQAS; Visiometrics, Terrassa, Spain) based on the principle of double pass using a laser diode wavelength of 780 nm. Measurements were taken after correcting the manifest refractive cylinder using an external lens and the machine automatically corrects the spherical error. The OSI was measured after 4–5 blinks and then at 0.5-second intervals over 20 s without the subject blinking in between.

The data was normally distributed and therefore the measured parameters were compared between both groups using the independent sample *t*-test. Statistical analysis was performed using commercial software (Stata ver. 12.1; StataCorp, College Station, TX). A *p* value of ≤ 0.05 was considered statistically significant.

## Results

Sixty eyes of 30 subjects with migraine (Group 1) and 60 eyes of 30 normal patients (Group 2) were enrolled in this study. There were 19 females and 11 males in each group. The mean age of subjects in Group 1 was (24.93 ± 4.54) years and that in Group 2 was (26.8 ± 6.16) years. Table 1 shows the comparison of aberrations and dry eye metrics between the two groups. Total aberrations (*p* = 0.049), higher order aberrations (*p* = 0.009), coma (p = 0.03), spherical aberrations (*p* = 0.018), Lipiview ICU (*p* < 0.001) and OSI (*p* < 0.001) were significantly different between the two groups. Trefoil (*p* = 0.26) and TBUT (*p* = 0.398) however, were not significantly different between both groups. The mean value of Schirmer’s test with anesthesia was (16.5 ± 6.4) mm in Group 1 and (17.4 ± 4.2) mm in Group 2, (*p* = 0.78). The mean corneal esthesiometry was similar between Group 1 (5.76 ± 0.4) cm and Group 2 (5.2 ± 0.8) cm, (*p* = 0.9).

**Table 1:** Aberrations (in microns) and dry eye evaluation data in eyes of migraine patients (Group 1) and controls (Group 2)

Parameter	Group 1 (Mean ± SD) (*N* = 60)	Group 2 (Mean ± SD) (*N* = 60)	*P* value
Total aberrations (RMS)	1.01 ± 0.34	1.20 ± 0.66	0.049*
Higher order aberrations (RMS)	0.38 ± 012	0.47 ± 0.03	0.009*
Coma (RMS)	0.20 ± 0.11	0.25 ± 0.13	0.030*
Trefoil (RMS)	0.23 ± 0.13	0.26 ± 0.16	0.260
Spherical aberration (RMS)	0.10 ± 0.06	0.16 ± 0.20	0.018*
Lipiview ICU	69.76 ± 5.20	63.18 ± 2.67	<0.001*
OSI	0.921 ± 0.14	1.26 ± 0.02	<0.001*
TBUT (seconds)	9.66 ± 1.51	9.41 ± 1.71	0.398

*RMS* = Root Mean Square; *ICU* = Interferometric Coloric Units; *OSI* = Ocular Scatter Index; *TBUT* = Tear film break up time

*n*: sample size

*indicates a statistically significant difference between the two groups. A *p*-value <0.05 was considered statistical significant

## Discussion

In this study, we looked at the correlation between measures of a dysfunctional tear film and migraine and compared it with controls. The trigeminal nerve is the common afferent for the migraine pathway as well as for dry eye associated ocular hyperalgesia and may play a role in the pathogenesis of ocular discomfort associated with migraine [10, 11].

Optical aberrations are a cumulative characteristic of the tear film, cornea and internal optics. It has been shown that the normal values of higher order aberrations may vary between various racial or ethnic groups [14]. Aberrations, in the presence of a healthy tear film are unlikely to change and are inherent to the patient’s optical system. The quality of vision can be influenced by inherent aberrations of the eye and/or those induced by alterations in the normal ocular structures like tear film abnormalities. All patient eyes included in our study had a SE of −0.50 diopters or less with a best-corrected distance visual acuity of 20/20 in each eye. However, the presence of significantly high aberrations and a dysfunctional tear film may play a role in degrading the quality of the retinal image, thereby acting as triggers for migraine. The association between dry eye and migraine has been investigated earlier [9, 15, 16]. However, in the current study, we did not include patients with Schirmer’s test value <10 mm/5 min thereby ruling out aqueous deficiency. This criteria for exclusion was applied because patients with frank dry eye will have increased aberrations on wavefront measurement due to a poor tear film layer, which in turn would lead to an erroneous overestimation of the individual’s aberration profile. TBUT though abnormal, was not significantly different in patient eyes compared to controls. Subjects and controls were age and gender matched as these factors themselves can be associated with dry eye disease. The reduced thickness of lipid layer causes the tear film to break up thereby causing the incident light to scatter as indicated by a significantly high OSI in eyes with migraine. Patient eyes were found to have statistically significant higher total, coma, spherical and higher order aberrations. Therefore, patients with migraine have inherently high aberrations with intra-ocular scatter and it is possible that a dysfunctional tear film may worsen the visual symptoms and be associated with migraineous attacks. An abnormal lipid layer as well as high aberrations may potentially act as triggers in predisposed patients. However, further studies with a larger cohort are required to establish this fact. To the best of our knowledge, the current study is the first study to study a possible association between ocular aberrations and migraine. Aberrometry thus has a potential role as a screening tool to identify patients with ocular triggers for migraine. This would also help classify patients who need aggressive management of borderline ocular surface dysfunction in an attempt to attenuate migraineous attacks in susceptible patients.

The statistical analysis in our study was performed eye wise rather than subject wise. We included both eyes of all subjects in both groups for analysis. This may have biased our results however, we feel that the inclusion of both eyes and respective relevant parameters is important and they would together contribute towards the genesis of a trigger. We also analyzed the data using one eye of each participant randomly and the results were not significantly different. The limitations of our study include need for a larger sample size. Also, the examination and measurements were performed in the non-ictal phase. Studying ictal phase tear film changes and aberrations will help us understand the potential role of these ocular factors in precipitating an attack. Also, a possible influence of the tear film dysfunction and aberrations on the duration, frequency and severity of migraneous attacks was not studied.

## Conclusions

We found that ocular aberrations were higher among patients with migraine as compared to controls and that ocular surface disease in migraine adds to the aberrations. This study demonstrates the association between chronic migraine and dysfunctional tear film along with ocular aberrations. Treating the ocular surface to obtain a healthy tear film might introduce a potential modifiable factor that will help reduce the frequency and severity of migraneous attacks. Studies with longer follow-up after treating the ocular surface will be needed to confirm our hypothesis.

## Abbreviations

ICU: Interferometric coloric units
OQAS: Optical quality analysis system
OSI: Objective scatter index
RMS: Root mean square
SE: Spherical equivalent
TBUT: Tear film break-up time
