# Correction to: The impact of dysfunctional tear films and optical aberrations on chronic migraine

**DOI:** 10.1186/s40662-017-0092-8

**Published:** 2017-11-13

**Authors:** Rohit Shetty, Kalyani Deshpande, Chaitra Jayadev, Kareeshma Wadia, Pooja Mehta, Rushad Shroff, Harsha L. Rao

**Affiliations:** Department of Ophthalmology, Narayana Nethralaya Eye Institute, 121/C, Chord Road, 1 “R” Block, Rajajinagar, Bangalore, Karnataka 560010 India

## Correction

After publication of this article [1] it came to our attention that Table 1 was presented incorrectly. The correct table can be found below:

**Table 1:** Aberrations (in microns) and dry eye evaluation data compared between migraine (Group 1) and controls (Group 2) using independent sample t- test

Parameter	Group 1 (Mean ± SD) (*N* = 60)	Group 2 (Mean ± SD) (*N* = 60)	*P* value
Total aberrations (RMS)	1.20 ± 0.66	1.01 ± 0.34	0.049*
Higher order aberrations (RMS)	0.47 ± 0.03	0.38 ± 0.12	0.009*
Coma (RMS)	0.25 ± 0.13	0.20 ± 0.11	0.030*
Trefoil (RMS)	0.26 ± 0.16	0.23 ± 0.13	0.260
Spherical aberration (RMS)	0.16 ± 0.20	0.10 ± 0.06	0.018*
Lipiview ICU	63.18 ± 2.67	69.76 ± 5.20	<0.001*
OSI	1.26 ± 0.02	0.921 ± 0.14	<0.001*
TBUT (seconds)	9.41 ± 1.71	9.66 ± 1.51	0.398

RMS = Root Mean Square; ICU = Interferometric Coloric Units; OSI = Ocular Scatter Index; TBUT = Tear film break up time

N: sample size

*indicates a statistically significant difference between the two groups. A *p* value < 0.05 was considered statistical significant
